# Electrostatic Tuning of the Ligand Binding Mechanism by Glu27 in Nitrophorin 7

**DOI:** 10.1038/s41598-018-29182-3

**Published:** 2018-07-18

**Authors:** Stefania Abbruzzetti, Alessandro Allegri, Axel Bidon-Chanal, Hideaki Ogata, Giancarlo Soavi, Giulio Cerullo, Stefano Bruno, Chiara Montali, F. Javier Luque, Cristiano Viappiani

**Affiliations:** 1 Dipartimento di Scienze Matematiche, Fisiche e Informatiche, Università degli Studi di Parma, Parco Area delle Scienze 7/A, 43124 Parma, Italy; 2 Department of Nutrition, Food Sciences and Gastronomy, Faculty of Pharmacy and Food Sciences and Institute of Biomedicine (IBUB), University of Barcelona, Avda. Prat de la Riba 171, Santa Coloma de Gramenet, Spain; 3 Max-Planck Institute for Chemical Energy Conversion, Stiftstrasse 34-36, D-45470 Mülheim an der Ruhr, Germany; 4 Cambridge Graphene Centre, University of Cambridge, 9 JJ Thomson Avenue, Cambridge, CB3 OFA UK; 5 IFN-CNR, Dipartimento di Fisica, Politecnico di Milano, Piazza Leonardo da Vinci 32, 20133 Milano, Italy; 6 Dipartimento di Scienze degli Alimenti e del Farmaco, Università degli Studi di Parma, Parco Area delle Scienze 27/A, 43124 Parma, Italy; 7 Present Address: Institute of Low Temperature Science, Hokkaido University Kita19-Nishi8, Kita-ku, 060-0819 Sapporo, Japan

## Abstract

Nitrophorins (NP) 1–7 are NO-carrying heme proteins found in the saliva of the blood-sucking insect *Rhodnius prolixus*. The isoform NP7 displays peculiar properties, such as an abnormally high isoelectric point, the ability to bind negatively charged membranes, and a strong pH sensitivity of NO affinity. A unique trait of NP7 is the presence of Glu in position 27, which is occupied by Val in other NPs. Glu27 appears to be important for tuning the heme properties, but its influence on the pH-dependent NO release mechanism, which is assisted by a conformational change in the AB loop, remains unexplored. Here, in order to gain insight into the functional role of Glu27, we examine the effect of Glu27 → Val and Glu27 → Gln mutations on the ligand binding kinetics using CO as a model. The results reveal that annihilation of the negative charge of Glu27 upon mutation reduces the pH sensitivity of the ligand binding rate, a process that in turn depends on the ionization of Asp32. We propose that Glu27 exerts a through-space electrostatic action on Asp32, which shifts the pKa of the latter amino acid towards more acidic values thus reducing the pH sensitivity of the transition between open and closed states.

## Introduction

Nitrophorins (NPs) comprise a unique class of ferrihemeproteins originating from the blood-feeding insect *Rhodnius prolixus*^1,2^. They participate in the storage, transport, and delivery of nitric oxide (NO) from the insect to the bite site, where NO acts as a vasodilator and as an anticoagulant to facilitate the feeding process. NO is bound at the axial position of the ferriheme centre in the acidic saliva of the insect (pH 5–6), enclosed in a cage formed by hydrophobic residues. The pH change experienced upon injection into the tissue of the insect’s victim (pH ~7.4 in the plasma) triggers a conformational transition from a closed to an open state that facilitates NO release^3,4^. NO release leads to vasodilation and inhibits platelet aggregation, thus benefiting the feeding process. Moreover, in the open conformation, NPs bind the imidazole group of histamine^5^, that mast cells release at the bite site as immune stimulus^6^, thus contributing to suppress the immune response during the feeding. Since NO is a pharmacologically relevant compound^7^, in order to foresee applications in cardiovascular diseases and cancer, it is important to understand the structural and dynamical properties of the proteins in relation to transport and release of the gaseous ligand.

**Figure 1:**
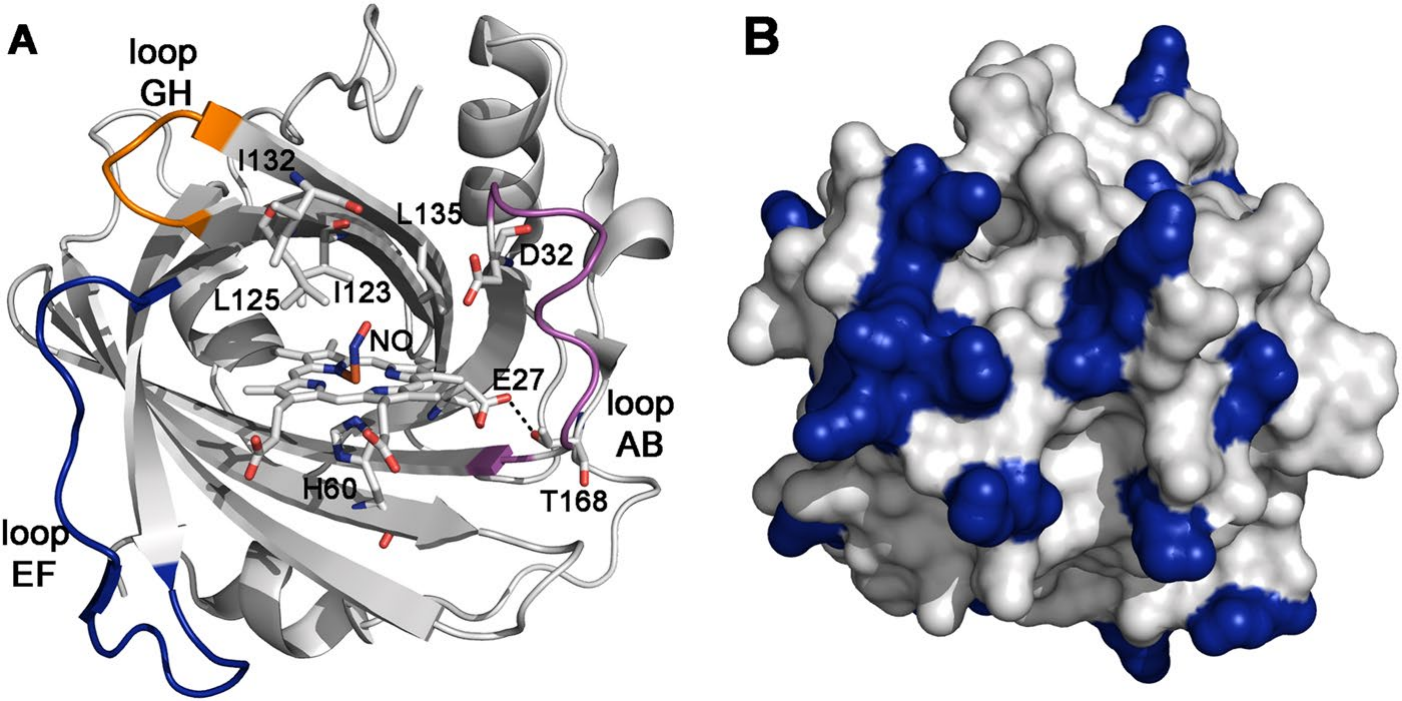
Key structural features of NP7. (**A**) Representation of the X-ray structure of NP7 from *Rhodnius prolixus* (PDB entry 4XME) showing the coordination of the heme to H60, the location of the residues (D32, I132) that form the hydrogen bond implicated in the conformational transition between open and closed states, and the position of E27, which is replaced by valine in other nitrophorins. Loops AB, EF, and GH are shown in magenta, blue, and orange, respectively. The hydrogen bond between E27 and T168 (distance: 2.8 Å) is shown as a dashed line. (**B**) Surface representation of NP7. Rear view opposite to the mouth of the heme cavity. Lysine residues are shown in blue.

The heme cofactor is located inside the central cavity of the lipocalin fold, which consists of an eight-stranded antiparallel β-barrel^8,9^. Coordinated to the proximal His60 (Fig. 1A), the heme iron is stable in the ferric form, whose NO affinity is low enough to make the release mechanism physiologically relevant. The pH-dependent NO release has been associated to the conformational remodeling of loops AB and GH^3,10–13^, a process related to the ionization state of Asp30 in NP4 (Asp32 in NP7; Fig. 1A), and the conversion between closed (low pH) and open (high pH) states. At acidic pH, Asp30 forms a hydrogen bond (HB) with the carbonyl group of Leu130 (Ile132 in NP7), keeping loops AB and GH close together. Deprotonation at high pH breaks the HB and the transition to the open state makes Asp30 to be solvent-exposed, favouring NO release to blood. The observed decrease in NP7 affinity for NO (from >9 to 6.6, expressed as log_10_ K_eq_) upon increasing the pH from 5.5 to 7.5 has been correlated with the closed ↔ open conformational transition^14^.

Although the pH-dependent release mechanism is also effective in NP1, NP2 and NP4, the high pH-sensitivity of NO affinity in NP7 suggests that some specific traits concur in this isoform, which is the only NP able to interact with L-α-phosphatidyl-L-serine -containing phospholipid membranes with high affinity (∼4.8 nM)^15,16^. This may be explained by the large cluster of Lys residues located at the protein surface opposite to the heme pocket (Fig. 1B), suggesting that NP7 may target the surfaces of activated platelets and degranulating mast cells. More intriguing is the presence of Glu27 in NP7 (Fig. 1A), which is strikingly replaced by Val24 in NP2 and NP3, or Val25 in NP1 and NP4. Being a unique trait of NP7, the functional implications of Glu27 remain to be elucidated. The X-ray structure of wild type (wt) NP7 shows that Glu27 adopts a folded conformation that facilitates the HB interaction with Thr168 (distance: 2.8 Å; Fig. 1A) both at pH 5.8 (aquo form; PDB ID 4XMC) and pH 7.8 (NO-bound complex; PDB ID 4XME)^9^. It has been suggested that the dense packing elicited by Glu27, together with Phe43, in the heme pocket affects the cofactor orientation inside the pocket^17^, and facilitates bond breakage between the heme and the proximal His in Fe(II) deoxy NP7^18^. However, keeping in mind the functional relevance of the conversion between closed and open states and the pH dependence of this conformational transition, the potential influence of Glu27 in modulating the kinetics of ligand binding emerges as a challenging question for understanding the role of NP7. In particular, it is unclear whether the negative charge of Glu27 may affect such conformational transition, and hence the accessibility of ligands to/from the heme pocket. To answer these questions, here we report the results of a comparative study performed on the wt NP7 and its E27Q and E27V mutants using a variety of experimental and theoretical techniques, including transient absorption (TA) spectroscopy with femtosecond to millisecond temporal coverage, X-ray crystallography and molecular dynamics (MD) simulations. Our results highlight the role of the negative charge of Glu27 in enhancing the pH sensitivity of ligand binding.

## Results and Discussion

### Kinetics of ligand rebinding after laser photolysis

**Figure 2:**
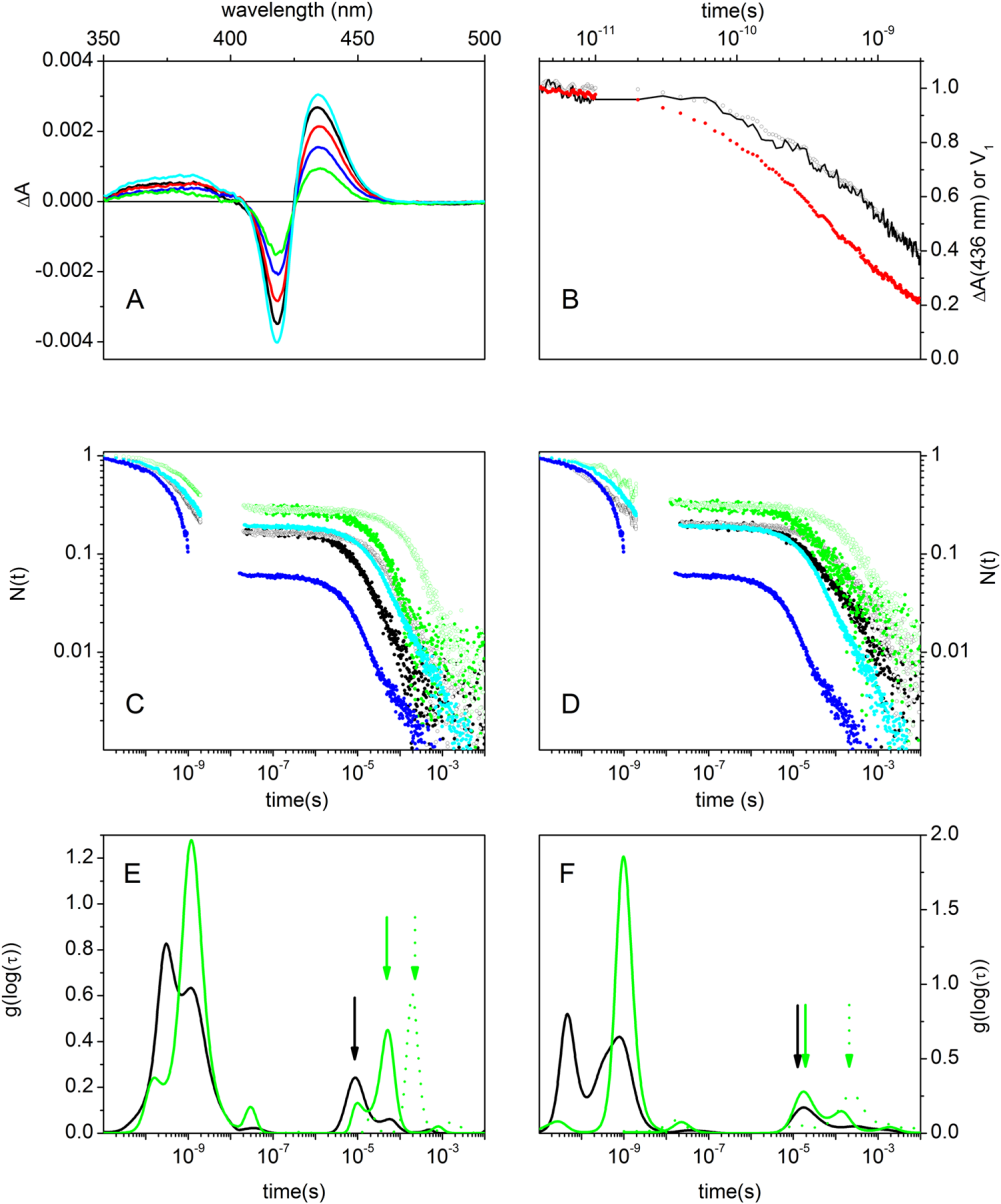
Ligand rebinding kinetics. (**A**) Time-dependent transient absorption spectra for CO-bound NP7(E27V) following femtosecond photoexcitation at 530 nm at 4 (black line), 200 (red line), 400 (green line) and 800 ps (blue line) delay times. First spectral component (U_1_S_1_ on a 1:10 scale) obtained from the SVD analysis of the time resolved differential absorption spectra multiplied by the corresponding singular value (S_1_ = 40.5) is shown as the cyan line. (**B**) Comparison between the time course of the amplitude *V*_1_ (black open circles) of the main spectral component obtained from SVD for CO-bound NP7 (E27V), and the normalized transient absorbance at 436 nm as a function of the delay time (black line). pH = 7.5, T = 20 °C. The amplitude *V*_1_ of the main spectral component obtained from SVD at pH 5.5 is reported as the red solid circles. Complete rebinding kinetics to (**C**) NP7(E27V) and (**D**) NP7(E27Q) at pH = 7.5 (green) and pH = 5.5 (black) reported as fraction of unliganded molecules vs time. The experimental progress curves were recorded at 1 CO atm (filled circles) and 0.1 CO atm (open circles). For comparison the data for CO rebinding to wt NP7 are reported in plots C and D at 1 atm CO at pH 5.5 (blue circles) and 7.5 (cyan circles).

Nanosecond laser flash photolysis and broadband femtosecond pump-probe spectroscopy were used to investigate the effect of Glu27 → Val/Gln mutations on the ligand rebinding kinetics. To this end, time-dependent TA spectra were obtained after femtosecond photolysis of the CO adducts of ferrous NP7(E27V) (see Fig. 2A for TA spectra collected at pH 7.5 at selected delays) and NP7(E27Q). CO was chosen as a model ligand for NP7[Fe(II)] in view of the amount of available literature data. Moreover, the reduced geminate yield for this ligand (see below) allows for an easier study of the bimolecular rebinding phase. The TA spectra, which correspond to the difference between the ground state absorption spectra of NP7[Fe(II)] and NP7-CO, display isosbestic points at 402 nm and 426 nm, indicative of a clean transformation between the two species. This was confirmed by singular value decomposition (SVD) analysis of the spectra collected between 10 ps and 2 ns, which delivered only one significant spectral component (cyan curve in Fig. 2A). Thus, the time course of the corresponding amplitude *V*_1_ (black open circles in Fig. 2B) perfectly matches the kinetics measured at 436 nm (black line in Fig. 2B). Similar results were obtained when the experiment was conducted at pH 5.5 and for the mutant NP7(E27Q) (data not shown).

Following a previously established procedure^9^, we merged the rebinding kinetics on the picosecond time scale with that measured in nanosecond laser flash photolysis experiments to obtain the overall rebinding curve for NP7(E27V) and NP7(E27Q) (Fig. 2C,D). For both mutants the sub-nanosecond kinetics becomes faster and of larger amplitude at pH 5.5, but the change is less prominent than for the wt protein^9^ and the related NP4^19^. On the longer time scales, the progress curves show features previously reported for the wt NP7, with a heterogeneous bimolecular rebinding^20^. However, the increase in the apparent rate of the bimolecular phase observed for wt NP7 as the pH is lowered to 5.5 is smaller for NP7(E27V) and becomes negligible for NP7(E27Q), indicating that annihilation of the negative charge of Glu27 upon mutation reduces the pH sensitivity of the ligand rebinding process.

These differences can be better appreciated in Fig. 2E,F, which show the (model independent) lifetime distributions associated with progress curves (Fig. 2C,D) determined using a Maximum Entropy Method. A total of 6 distinct bands are evident in the lifetime distributions. Three bands are present in the ps-ns time range, accounting for fast geminate rebinding. When the pH is lowered to 5.5, the band at ~10^−10^ s (~5 × 10^−11^ s for E27Q) becomes larger at the expense of the band at ~10^−9^ s, in agreement with the larger and faster geminate rebinding seen in Fig. 2C,D. In the μs-ms range three bands can be recognized. The band marked with an arrow is due to bimolecular rebinding, as deduced by the shift in peak position between 1 atm CO (solid green) and 0.1 atm CO (dotted green) at pH 7.5. When the pH is lowered to 5.5, the bimolecular rebinding rate for NP(E27V) at 1 atm CO (Fig. 2E) increases, whereas for NP(E27Q) (Fig. 2F) this rate remains essentially unchanged (green and black solid arrows). An additional unimolecular step is present at about 10 μs, as noted from the comparison between the 0.1 and 1 atm CO distributions. Finally, the band in the 2 ms range is scarcely affected by pH and CO concentration and may be attributed to a relaxed conformation populated in the deoxy molecule^20^.

For NP7(E27V) the amplitude of the sub-nanosecond geminate rebinding increases from 0.72 at pH 7.5 to 0.85 at pH 5.5, as expected from the enclosure of the ligand into the hydrophobic cage formed by Ile123, Leu125, Ile132 and Leu135 in the heme cavity (Fig. 1A). A similar increase from 0.70 at pH 7.5 to 0.80 at pH 5.5 is observed for NP7(E27Q). Remarkably, the amplitudes for wt NP7 were 0.83 and 0.95 at pH 7.5 and 5.5, respectively, thus revealing a larger geminate rebinding, especially at acidic pH. This suggests that the Glu27 → Val/Gln mutations likely perturb the structure of the heme cavity, altering the spatial orientation of the residues that form the hydrophobic cage around the heme-bound ligand.

**Figure 3:**
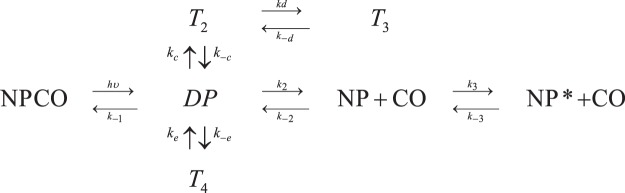
Minimal reaction scheme for the observed rebinding kinetics.

Figure 3 shows the microscopic model that was proposed for the ligand rebinding kinetics in wt NP7 according to the topology of inner cavities^9^. After photolysis (hν), the ligand is found in the distal pocket (DP) from which it can be rebound (rate *k*_−1_), or migrate to cavities located either in the back tunnel of the protein (*T*_2_, rates *k*_±c_ and *T*_3_, rates *k*_±d_)^9^, or next to the heme distal pocket (*T*_4_, rates *k*_±e_). Alternatively, the ligand can exit from DP to the solvent (this intermediate is indicated as NP, rate *k*_+2_), from which the ligand can be rebound in a bimolecular reaction (rate *k*_−2_). Finally, relaxation to a slowly reactive species (NP*) occurs from the deoxy species (NP). The corresponding microscopic rate constants determined for NP7(E27V) and NP7(E27Q) are reported in Table 1 (a comparison of the global rebinding kinetics analysis to the wt NP7 and its mutated variants is shown in Supporting Information Fig. S1).

**Table 1:** Microscopic rate constants from the global fit of the CO rebinding kinetics at 20 °C.

Rate constants	pH = 7.5			pH = 5.5		
NP7	wt	E27V	E27Q	wt	E27V	E27Q
*k*_−1_ (10^10^ s^−1^)	**0.60 ± 0.02**	**0.2 ± 0.01**	**0.6 ± 0.2**	**1.4 ± 0.04**	**0.32 ± 0.01**	**0.70 ± 0.2**
*k*_2_ (10^8^ s^−1^)	9.0 ± 0.4	6.4 ± 0.3	15 ± 6	8.2 ± 0.4	5.6 ± 0.3	10 ± 2
*k*_−2_ (10^8^ M^−1^ s^−1^)	0.6 ± 0.1	0.6 ± 0.1	0.9 ± 0.1	1.6 ± 0.2	1.3 ± 0.2	1.0 ± 0.1
*k*_3_ (10^3^ s^−1^)	**2.0 ± 0.5**	**1.7 ± 0.4**	**20 ± 6**	**2.9 ± 0.7**	**2.4 ± 0.6**	**21 ± 1**
*k*_−3_ (10^3^ s^−1^)	1.2 ± 0.5	1.9 ± 0.8	3 ± 1	4 ± 1	1.2 ± 0.5	3 ± 1
*k*_*c*_ (10^9^ s^−1^)	3.7 ± 0.5	2.9 ± 0.4	1.7 ± 0.8	0.5 ± 0.1	2.3 ± 0.3	1.3 ± 0.8
*k*_*−c*_ (10^9^ s^−1^)	**1.4 ± 0.2**	**1.9 ± 0.2**	**0.07 ± 0.01**	**1.2 ± 0.1**	**2.4 ± 0.3**	**0.07 ± 0.01**
*k*_*d*_ (10^8^ s^−1^)	1.6 ± 0.1	1.8 ± 0.1	1.01 ± 0.06	2.1 ± 0.1	0.93 ± 0.05	1.01 ± 0.06
*k*_*−d*_ (10^4^ s^−1^)	1.4 ± 0.4	1.4 ± 0.4	4.9 ± 1	1.2 ± 0.3	1.2 ± 0.4	5 ± 1
*k*_*e*_ (10^10^ s^−1^)	4.5 ± 0.7	7 ± 1	3 ± 1	8.0 ± 1	3.7 ± 0.6	1 ± 1
*k*_*−e*_ (10^10^ s^−1^)	2.5 ± 0.3	6.5 ± 0.7	0.6 ± 0.1	1.9 ± 0.2	9 ± 1	0.6 ± 0.1
*k*_*on*_ (10^8^ M^−1^ s^−1^)	**0.5 ± 0.1**	**0.5 ± 0.1**	**0.7 ± 0.1**	**1.5 ± 0.3**	**1.1 ± 0.2**	**0.7 ± 0.1**
NO-Fe(III)^§^						
*k*_*on*_ (10^6^ M^−1^ s^−1^)	2.4			N.A.		
*log(K*_*eq*_ *(M* ^*−*1^ *))*	6.6 ± 0.1			>9		
**NP4**						
Fe(II)-CO^#^						
*k*_*on*_ (10^8^ M^−1^ s^−1^)	0.19/0.45			0.37		
Fe(III)-NO^&^						
*k*_*on*_ (10^6^ M^−1^ s^−1^)	2.3*			2.1		
*log(K*_*eq*_ *(M* ^*−*1^ *))*	6.92*			7.3		

Literature *k*_*on*_ values for NO binding to Fe(III) NP7 and Fe(III) NP4 and for CO binding to Fe(II) NP4 are reported for comparison. Values in bold denote kinetic parameters that undergo relevant changes between the distinct NP7 species. ^§^Data for NO binding to Fe(III) NP7 are from ref.^14^. ^#^Data for CO binding to Fe(II) NP4 are from ref.^20^. ^&^Data for NO binding to Fe(III) NP4 are from ref.^4^. *Values determined at pH = 8.0.

Inspection of the parameters in Table 1 shows that while the overall rebinding rate constant *k*_*on*_ at pH 7.5 is similar for the wt protein and the mutants (0.5–0.7 × 10^8^ M^−1^ s^−1^), the 3-fold increase in *k*_*on*_ observed for wt NP7 at pH 5.5, where the closed species should be the major form, is not paralleled in the case of NP7(E27V), for which the rate increases only 2-fold, or NP7(E27Q), for which *k*_*on*_ is essentially pH independent. This trend agrees with the changes in pH sensitivity of the binding rate *k*_−1_, as the increase (~2.3-fold) in *k*_−1_ observed for wt NP7 upon reduction of pH from 7.5 to 5.5 is progressively lost for the two mutants. It is worth observing that for NP4, where Val25 takes the place of Glu27 in NP7, the change in *k*_*on*_ between pH 5.5 and 7.5 is almost negligible^20^, a fact that is true also for NO binding to the ferric NP4^4^ (see Table 1). The small change in the equilibrium NO binding constant for NP4 thus arises solely from the dissociation rate constant. Similar considerations apply to NP1^4^. In the case of NP7, the 3-fold change in *k*_*on*_ is nevertheless not sufficient to explain the very large variation in affinity with pH^14^, therefore a major contribution from the dissociation rate constant is expected.

Finally, we observe that the largest changes affect the rate constants *k*_*−c*_ and *k*_3_, which vary by a factor close to 0.05 and 10, respectively, upon mutation of Glu27 to Gln. The rate constant *k*_*−c*_ corresponds to ligand detrapping from the inner docking site T_2_ (Fig. 3), and the smaller value obtained for the NP7(E27Q) mutant suggests that the gaseous ligand may be stabilized in the interior of the β-barrel. On the other hand, the rate constant *k*_3_ measures the relaxation from the unbound conformation of NP7 to the slowly reactive one (NP* in Fig. 3). The increase in *k*_3_ obtained for NP7(E27Q) suggests that the mutation may alter the structure of the heme cavity, possibly facilitating the access of water molecules that may reside in the distal pocket^21^ or bind the heme cofactor, as observed for the Fe(III) species^18^.

Overall, these results suggest that mutation of Glu27 to Val/Gln has a significant influence on the rebinding kinetics. This is an unexpected finding, as Glu27 does not participate directly in the hydrophobic cage that surrounds the ligand nor in the shaping of the inner cavities in the lipocalin fold.

### X-ray structures of NP7 and NP7(E27V)

The X-ray structure of NP7(E27V) was solved in order to examine the impact of the Glu27 mutation on the structure of the heme cavity. Data collection and refinement statistics of two X-ray structures solved for NP7(E27V) at pH 6.8, the aquo form and the complex with heme-bound imidazole (IMH), are provided in Supporting Information Table S1. Unfortunately, attempts to obtain the X-ray structure of NP7(E27Q) were unsuccessful due to lower solubility and stability of the protein.

**Figure 4:**
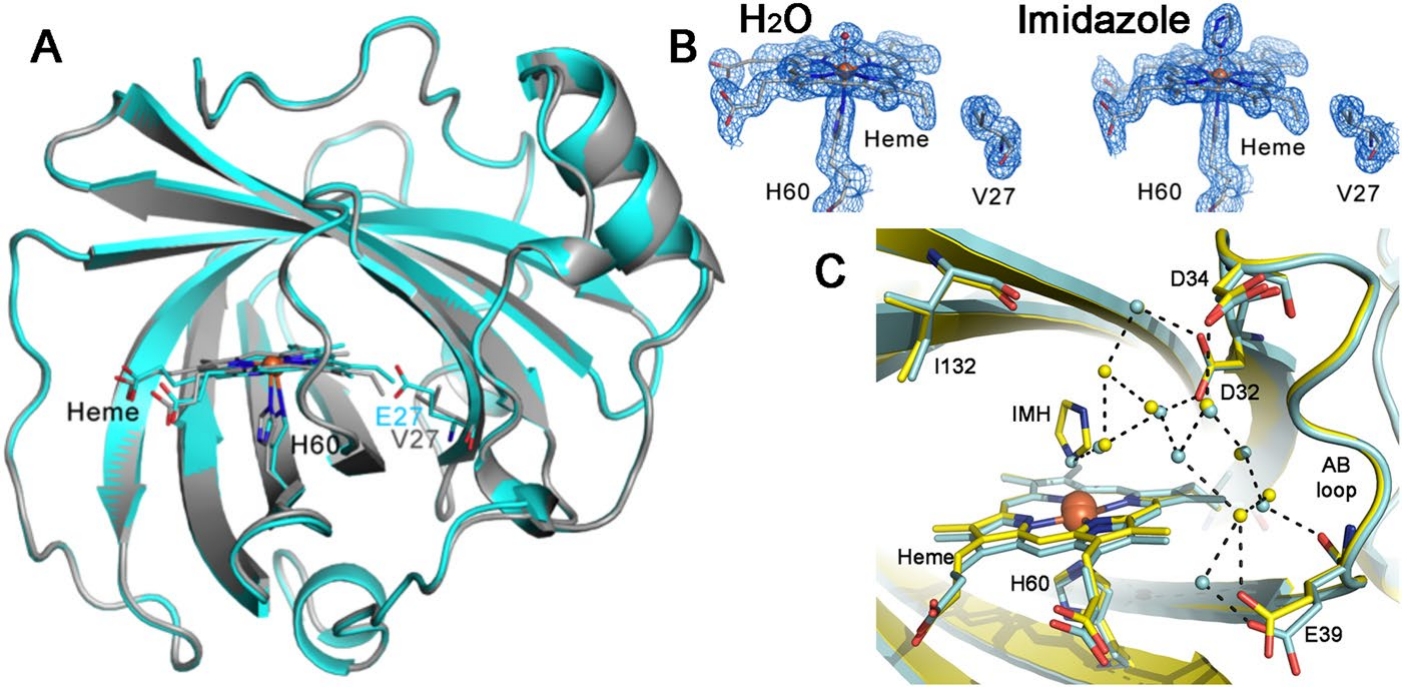
X-ray structure of Np7(E27V). Superposition of the X-ray structure of wt NP7 (PDB entry 4XMC; grey) and the aquo form of NP7(E27V) (PDB entry 5M6J; cyan). (**B**) Electron density maps (2Fo-Fc; contoured at 1σ) of the heme in (*left*) NP7(E27V) and (*right*) NP7(E27V)ImH. (**C**) Local structural details of the heme pocket in NP7(E27V) (cyan) and NP7(E27V)ImH (yellow) after superposition of the protein backbone. Selected residues and water molecules are shown as sticks and spheres, respectively. The network of hydrogen bonds is shown as dashed lines.

The overall fold of NP7(E27V) closely resembles the 3D skeleton of wt NP7 (Fig. 4A), as noted in the Root Mean Square Deviation (RMSD) values for the protein backbone (upon exclusion of the first three and last four residues) close to 0.2 Å for the two NP7(E27V) structures relative to the wt NP7. The AB and GH loops in NP7(E27V) closely fit the arrangement found in wt NP7 solved at both pH 5.8 and 7.8^18^. However, this can be explained by the reduced conformational flexibility imposed by the head-to-tail arrangement of proteins in the crystal lattice, which involves numerous salt bridges between the clustering of Lys residues at the rear surface of the protein (Fig. 1B), and the negative charges located at the heme mouth.

The electron density of the cofactor in NP7(E27V) is well defined (Fig. 4B), indicating that the heme is in the **B** orientation (Supporting Information Fig. S2), as found in other NPs for which Val replaces Glu^22,23^. It also superposes well the heme in the X-ray structure of wt NP7 (Fig. 4C), which also contains **B**-heme due to packing effects in the crystal lattice^9^, since NMR studies demonstrated the reversal to the **A** orientation in aqueous solution^17^. In contrast, NMR studies showed that NP7(E27V) mainly contains **B**-heme, although a significant fraction of **A**-heme was also found (ratio **A**:**B** of 1:3 at pH 5.5), thus revealing some degree of heme rotational disorder^17^. Importantly, replacement of Glu27 by Gln in wt NP7 did not change the heme **A** orientation, but replacement of Val24 by Glu in NP2 resulted in the **B** → **A** reorientation of the cofactor^24^. Thus, the steric hindrance provided by the glutamate residue is responsible for the **A** heme orientation rather than the carboxylate charge^17^.

Finally, in NP7(E27V) Asp32 is not hydrogen-bonded to Ile132 (i.e., the interaction that would mimic the Asp30-Leu130 HB in NP4), but participates in a dense network of water-mediated bridges with Asp34, Glu39, and the ligand in the distal side (Fig. 4C). Note that breakage of the Asp32-Ile132 HB was found even at the acidic conditions used in crystallization (pH 5.5), as was also found for the X-ray structure of wt NP7^9^, suggesting that this structural feature likely reflects the disturbing effect caused by the head-to-tail electrostatic interactions in the crystal.

### Molecular dynamics of NP7(E27Q) and NP7(E27V) variants

The close resemblance between the X-ray structures of wt NP7 and NP7(E27V) may be misleading for interpreting the functional behavior of NP7 in solution, as key structural details, such as the heme orientation, the spatial layout of the AB loop, and the absence of a HB between Asp32 and Ile132 may be affected by the packing. Therefore, MD simulations were run to explore the structural properties of the closed state of ligand-bound NP7(E27Q) and NP7(E27V). For NP7(E27V) simulations were run modeling the heme in both **A** and **B** arrangements, which are found in aqueous solution (see above), and simulations of NP7(E27Q) were run with the **A**-heme, which is the most favored orientation in solution^16^. To this end, two distinct starting arrangements of Gln27 that differ in the orientation of the sidechain amide group (denoted Q1 and Q2 hereafter) were considered. In these orientations, the amide group of Gln27 was superposed onto the sidechain of Glu27 found in the X-ray structure, and the relative position of the amide O and N atoms were exchanged, while preserving the HB formed with Thr168.

**Figure 5:**
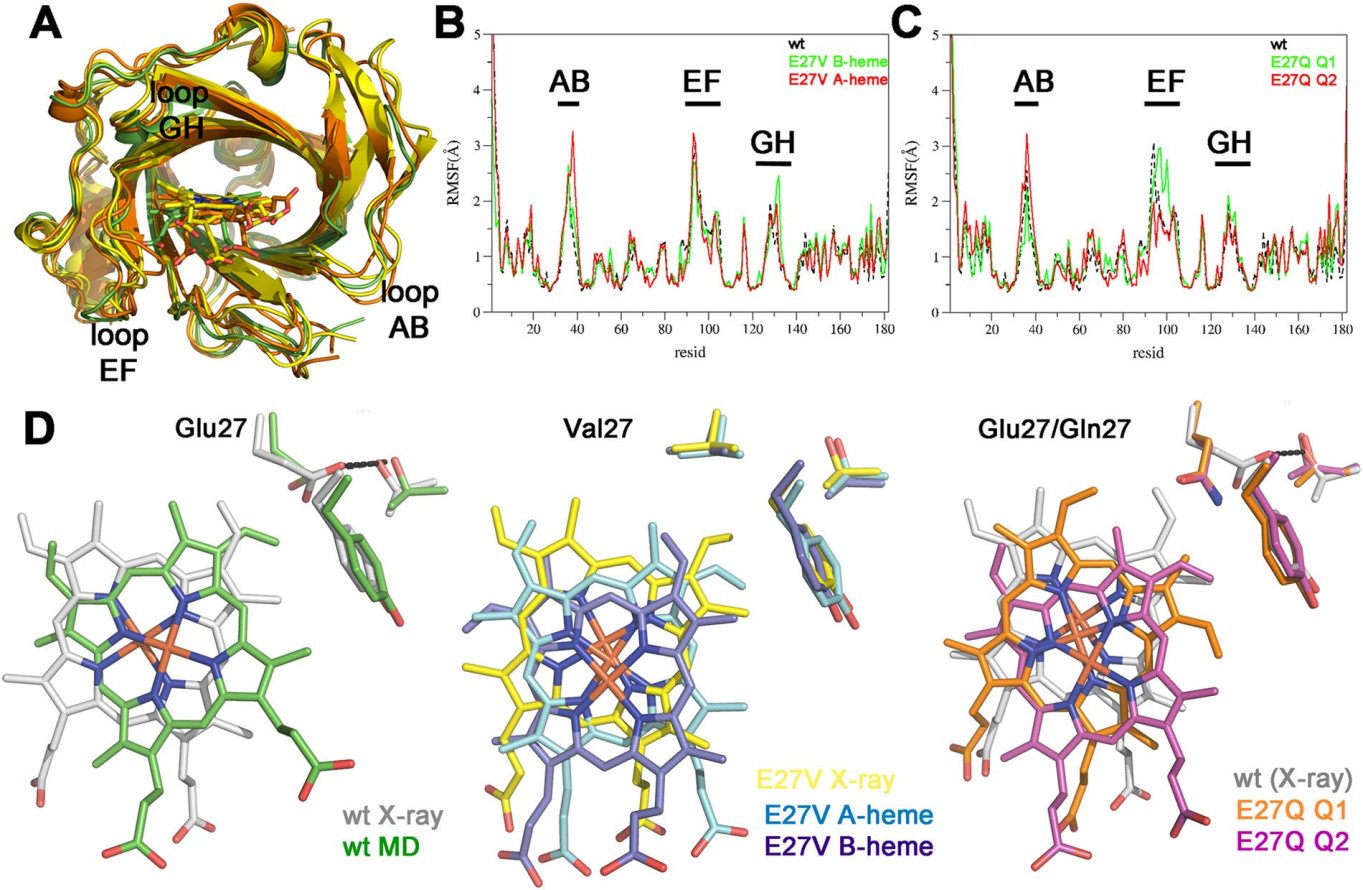
Structural analysis of simulated proteins. (**A**) Superposition of the energy-minimized averaged structures collected from the last 20 ns of MD simulations run for the closed forms of wt NP7 (green) and the E27V (**A**- and **B**-heme; yellow) and E27Q (Q1 and Q2; orange) variants. The heme group is shown as sticks. (**B**,**C**) RMSF (Å) profile by residue (excluding hydrogen atoms) for wt NP7 (dashed black) and its mutated E27V (**B**-heme: green; **A**-heme: red) and E27Q (Q1: green; Q2: red) variants. (**D**) Position of the heme and selected residues (Glu27, Thr168, and Tyr30) after superposition of the protein backbone in the X-ray and MD energy-minimized averaged structures of wt NP7 and its E27V and E27Q mutated variants. Hydrogen bonds are shown as dashed lines.

The MD averaged structures of the NP7(E27V) and NP7(E27Q) are highly similar (Fig. 5A), as noted in RMSD values for the backbone atoms (excluding the first three and four residues) in the range 1.4–1.9 Å, which is reduced to 1.0–1.2 Å upon exclusion of loops AB, EF and GH (residues 31–41, 91–104, and 127–135; Supporting Information Table S2), which are the most flexible regions. In fact, the pattern of root-mean square fluctuations (RMSF) along the sequence is highly similar for both wt and mutated proteins, showing only slight local differences, such as the larger fluctuations of loops AB and EF found for NP7(E27V) (Fig. 5B) and NP7(E27Q2) (Fig. 5C) variants, respectively. For the sake of completeness, comparison with the averaged structure of the open form of the wt NP7 (WTo in Table S2), shows much larger RMSD values (between 2.3 and 3.1 Å), even after exclusion of loops AB, EF and GH (RMSD of 1.7–2.0 Å).

While the global conformation of the β-barrel is well preserved along the trajectories run for wt NP7 and its mutated variants, there are local differences in the heme cavity (Fig. 5D). Compared to the X-ray structure of wt NP7, the iron atom in the simulated wt NP7 is displaced by 1.2 Å, and the heme is anti-clockwise rotated by 45 degrees, presumably to reduce the steric clash that would occur between a vinyl group and Tyr30 if the tetrapyrrole core of the **A**-heme in the MD simulation superposed perfectly the **B**-heme in the X-ray structure. Rotation of the **A**-heme alleviates the steric hindrance, this geometrical change being further assisted by the folded conformation of Glu27, which forms a HB with Thr168. In NP7(E27V) the heme is displaced toward the mouth of the heme pocket, mimicking the shift observed in the wt NP7, but without showing a significant rotation (<16 degrees) within the heme pocket. These changes are similarly found in the simulations run for the heme in **A** and **B** orientations, reflecting the reduced steric hindrance originated from the E27V mutation. In contrast, distinct orientations that differ in a rotation of ~50 degrees are found for the **A**-heme in the two simulations run for the E27Q variant. Although the initial structures of the E27Q mutants were built up to maintain the HB with Thr168, this interaction was lost along the two trajectories, and Gln27 adopted an extended conformation, while promoting distinct rotations of the heme relative to the X-ray structure of wt NP7. Let us note that the rearrangements observed in the heme occurred at the beginning of the trajectory and remained stable along the simulation, showing only small fluctuations around the average structure shown in Fig. 5D (see also Supporting Information Fig. S3). In turn, these changes also affected the cage-like arrangement of the hydrophobic residues that enclose the heme-bound ligand, especially regarding the spatial arrangement of Leu125 and Ile132 (Supporting Information Fig. S4). Overall, the notable structural differences in the heme cavity found between wt NP7 and its mutated variants reveal the steric tension acting on the cofactor, which seems to be partly alleviated by the HB between Glu27 and Thr168 in wt NP7.

### pKa computations of Asp32

The local structural alterations exerted by the Glu27 → Val/Gln mutation have different consequences on the heme orientation, mostly due to distinct steric hindrance. In addition, annihilation of the negative charge of Glu27 may have an influence on the transition between open and closed states. Since this process has been associated to the deprotonation of Asp30 in NP4^3,4^, can the Glu → Val/Gln mutation affect the microscopic pKa of Asp32 in NP7?

The experimentally observed apparent pKa of 6.5 in NP4^23^ has been interpreted by combining the conformational equilibrium between closed and open species, and the microscopic pKa of Asp30 in these states, which was estimated to vary from 4.3 in the open species to 8.5 in the closed protein according to constant pH MD simulations^13^. Adaptive Poisson Boltzmann Solver (APBS) calculations performed for a set of snapshots of the open wt NP7 (taken from ref.^25^) indicated that the microscopic pKa of Asp32 is shifted by only 0.1 pKa units relative to the standard pKa (i.e., the pKa of the fully solvent-exposed carboxylate group in Asp; pKa of 4.0), which matches the value reported for Asp30 in the open form of NP4 (pKa of 4.3)^13^. This can be realized by the solvent exposure of Asp32 to water molecules, and hence the maintenance of the standard pKa of Asp in the open form of NP7, which can be assumed to be also the microscopic pKa of Asp32 in the open forms of NP7(E27V) and NP7(E27Q). Indeed, a similar shift (pKa of 4.2) was found for the snapshots collected for the open form of E27V NP7 from an additional MD simulation (data not shown), which was performed following the procedure described for the wt NP7^25^.

The corresponding pKa predicted for the closed state of NP4 amounts to 8.9, which agrees with the pKa of 8.5 reported in previous studies^13^. However, a much larger shift is predicted for the closed species of NP7, as the microscopic pKa is estimated to be 6.9. Compared to NP4, this shift can be ascribed to the large number of positively charged residues in wt NP7, as reflected in the larger pI value (close to 9) relative to NP1-NP4 (pI of 6.1–6.5). Remarkably, mutation of Glu27 to Val and Gln gives rise to an additional reduction in the microscopic pKa of Asp32, which is predicted to be 6.3 and 5.7, respectively. This can be rationalized from the repulsive electrostatic interaction between Glu27 and the ionized form of Asp32 in the wt NP7, which would tend to destabilize the ionized closed species with regard to both NP7(E27V) and NP7(E27Q).

At this point, let us note that APBS calculations predict Glu27 to be fully ionized in the closed form of wt NP7 (pKa ~ 2.0). This can be qualitatively explained by the stabilization afforded by hydrogen-bond interactions formed with the hydroxyl group of Thr168, the backbone NH group of Phe43, and the hydroxyl group of Tyr175, as well as by the electrostatic interaction afforded by Lys143, after rearrangement of its side chain along the MD simulation. Compared to the X-ray structure (PDB code 4XMC), these interactions only require a slight rearrangement of the side chain of Glu27 in the heme pocket (see Supporting Information, Fig. S5). The anionic character of Glu27 is also maintained in the open state (pKa ~ 3.1). In this case Glu27 adopts an extended conformation (see Supporting Information, Fig. S6), which arises from the relief of the steric stress in the closed NP7 triggered by the ‘opening’ of the loop AB, which makes the heme cavity more accessible to bulk solvent.

**Figure 6:**
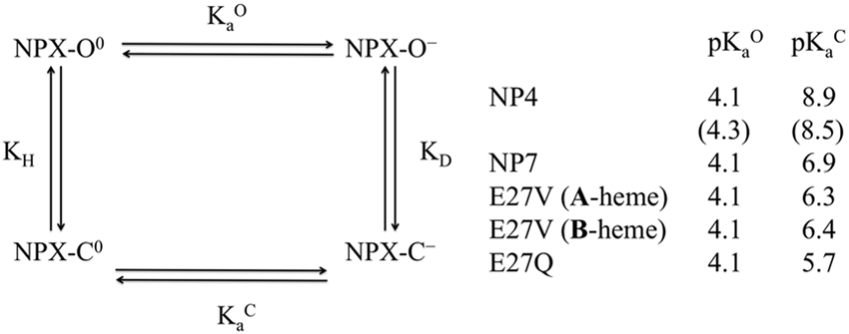
Thermodynamic cycle that relates the ionization state of Asp32 in NP7 and Asp30 in NP4 with the conformational transition between open and closed states of the proteins. The protonated and ionized forms of Asp32/Asp30 are indicated with the superscripts 0 and −, and the open and closed conformations are denoted with the superscripts O and C. K_a_^o^ and K_a_^C^ denote the ionization constants of Asp32/Asp30 in the open and closed states, and K_D_ and K_H_ stand for the conformational equilibrium constant in the dissociated and protonated forms. The pK_a_^o^ and pK_a_^C^ values were determined from APBS computations (the pKa values reported in ref.^11^ are given in parenthesis).

The thermodynamic cycle shown in Fig. 6 can be used to relate the ionization of Asp32 and the closed ↔ open conformational transition of the protein^13^. According to this cycle, the ratio between the conformational constants $\frac{{K}_{D}}{{K}_{H}}$ must equal the ratio between the ionization constants $\frac{{K}_{a}^{O}}{{K}_{a}^{C}}$ (see Fig. 6 for definition of equilibrium constants). From the calculated pKa values, this ratio decreases from a factor close to 68,000 (16,000 from the pKa values in ref.^10^) for NP4, to 630 for wt NP7, and to 190 and 40 for NP7(E27V) and NP7(E27Q), respectively. Thus, compared to NP4, the conformational transition between closed and open species in NP7 and its mutated variants is less influenced by the ionization state of Asp32.

In the case of NP4, *K*_*D*_ was estimated to be ~100^13^, indicating that the conformational equilibrium is displaced toward the open, ionized species (NP4-O^−^) at high pH, and to the closed, protonated species (NP4-C^0^) at low pH (Fig. 6). This agrees with the pH dependence of the CO geminate rebinding, which increased from 0.7 at pH 7 to 0.95 at pH 5^19^, reflecting the predominance of the NP4-O^−^ and NP4-C^0^ species at these pH values. The apparent pKa for the transition between open and closed states can be estimated from Eq. 1^13^, yielding an apparent pKa of 6.9, which compares with the experimental value of 6.5^22^.

1 $${\rm{apparent}}\,{\rm{pKa}}={{{\rm{pK}}}_{{\rm{a}}}}^{{\rm{O}}}+{{{\rm{pK}}}_{{\rm{a}}}}^{{\rm{C}}}-\,\mathrm{log}(\frac{{{\rm{K}}}_{{\rm{D}}}+{\rm{1}}}{{{\rm{K}}}_{{\rm{D}}}{{{\rm{K}}}_{{\rm{a}}}}^{{\rm{C}}}+{{{\rm{K}}}_{{\rm{a}}}}^{{\rm{O}}}})$$

Furthermore, the population of the protonated and deprotonated forms of closed and open species of NP4 (see Supporting Information Table S3) indicates that the protonated closed species (NP4-C^0^) is the major form (96% population) at pH 5.5, whereas the ionized open form (NP4-O^−^) is the most populated species (79.3%) at pH 7.5, although there is a minor fraction (19.9%) of NP4-C^0^. This agrees with the single phase observed for the bimolecular CO rebinding (*k*_on_ = 3.7 × 10^7^ M^−1^ s^−1^) at pH 5.5, and the heterogeneous rebinding kinetics at pH 7.5 (with *k*_on_ values of 4.5 × 10^7^ M^−1^ s^−1^ (27%) and 1.9 × 10^7^ M^−1^ s^−1^ (73%))^20^. Accordingly, one may assume the correspondence between the closed ↔ open transition and the ionization of Asp30, since the protein with protonated (NP4-C^0^) and ionized (NP4^−^O^−^) Asp30 should be largely representative of the conformational ensemble populated at acidic and neutral pH, respectively.

**Figure 7:**
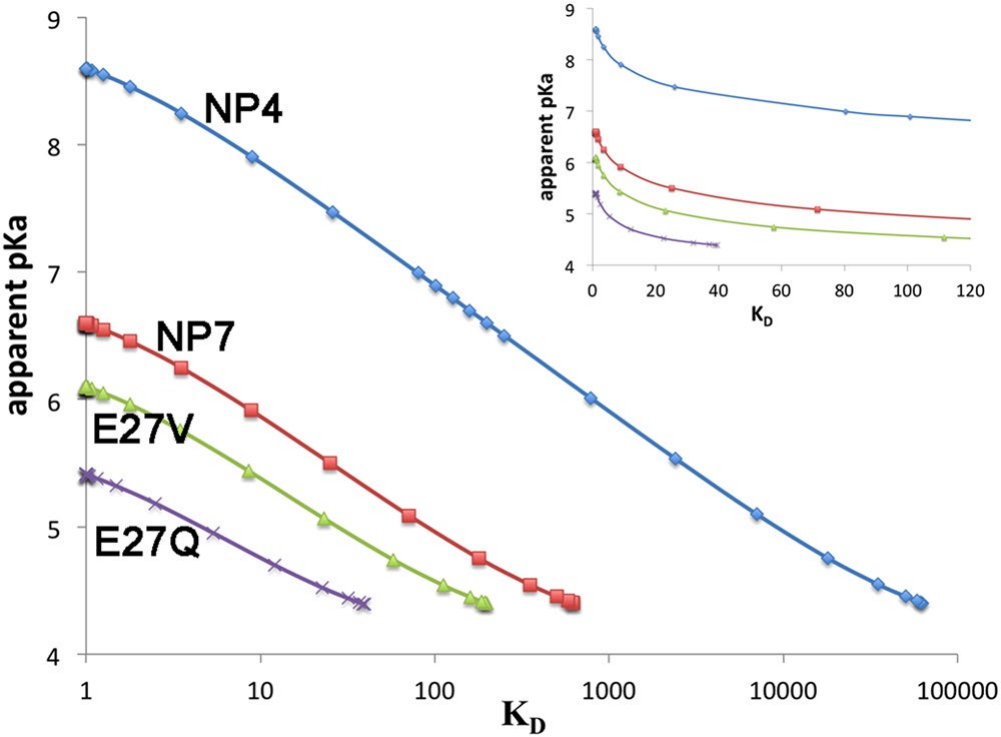
Dependence of the apparent pKa of wt NP4 and NP7, and the mutated variants NP7(E27V) and NP7(E27Q) on the equilibrium constant (K_D_) for the conversion between the closed and open forms of the ionized protein.

To the best of our knowledge, no quantitative estimate of *K*_*D*_ is available for wt NP7. If we assume that *K*_*D*_ = 100, the protein should be largely insensitive to pH, since the open species should be the major species at both pH 5.5 (80%, with 76.9% corresponding to the ionized form, NP7-O^−^) and 7.5 (98.7%, NP7-C^0^), which seems to be counterintuitive. Rather, the dependence between the apparent pKa and *K*_*D*_ (Fig. 7) reveals that the apparent pKa for the transition between closed and open species must be lower than 6.5 (i.e., the apparent pKa for NP4), this effect being even larger for the mutated proteins^23^. This would justify the reduction in pH sensitivity of NP7(E27V) and NP7(E27Q) to bimolecular ligand binding, reflecting the larger stabilization of the closed species NP7-C^−^ in the mutated proteins due to the electrostatic destabilization between Glu27 and ionized Asp32 in wt NP7.

Keeping in mind that the ratio $\frac{{K}_{D}}{{K}_{H}}$ is 630 for wt NP7, and assuming that the conformational equilibrium between neutral species (NP7-O^0^, NP7-C^0^) is not altered by the excess positive charge present in NP7, *K*_*D*_ can be estimated to be ∼4. Under these conditions, the population of the closed species should be 86.3% at pH 5.5 (83.3% corresponding to NP7-C^0^), whereas at pH 7.5 the ionized open form (NP4-O^−^) would predominate (76.2%), and the population of the ionized closed species (NP4-C^−^) would be close to 19.0%. Therefore, the reduction in *K*_*D*_ would enable the recovery of the pH sensitivity in the wt NP7, thus mimicking the behavior found for NP4. Moreover, the mutated proteins would be even less sensitive to changes in the environmental pH: the predicted populations of the open species would be 31.7% and 78.7% at pH 5.5 and 7.5, respectively, for NP7(E27V), and 61.7% (pH 5.5) and 79.7% (pH 7.5) for NP7(E27Q).

Within the uncertainties of pKa calculations, these findings provide a basis to explain the different second-order rebinding kinetics observed experimentally for wt NP7 in solution at pH 7.5 and 5.5, which partly accounts for the dramatic increase in ligand affinity at low pH. Furthermore, they are in agreement with the progressive decrease in the ratio between the *k*_*on*_ rate constants determined at pH 5.5 and 7.5, which decreases from 3 to 2.2 to 1.0 for wt NP7, NP7(E27V) and NP7(E27Q), respectively (Table 1).

### Conclusions

This study allow us to conclude that Glu27 has a relevant effect on the pH sensitivity of ligand binding for wt NP7. When the pH is lowered from 7.5 (open conformation, for wt NP7) to 5.5 (closed conformation), a smaller change in the ligand binding rate *k*_on_ (and *k*_−1_) is observed for NP7(E27V), while no change is seen for NP7(E27Q), in comparison with wt NP7. Thus, replacement of the negatively charged Glu27 with neutral residues results in progressive loss of the characteristic pH sensitivity of the ligand binding rate observed for the wt protein.

In order to explain the observed effects on the ligand binding rate, we propose that the negative charge of Glu27, and hence the electrostatic repulsion with deprotonated Asp32, introduces a mechanism, yet to be identified in detail, to modulate the pH sensitivity of NP7 and the apparent pKa for the transition between the closed and open states, characterized by high and low binding rates, respectively. This is readily explained by a thermodynamic equilibrium where open and closed conformations are linked to protonation of Asp32. In the presence of a neutral residue at position 27, NP7 would have been weakly sensitive to pH changes, as the excess positive charge of the protein would have stabilized the ensemble of closed conformations with deprotonated Asp32.

It remains to be understood if this fine tuning of the pH sensitivity may be intertwined with the unique ability of NP7 to target negatively charged membranes with high affinity through the interaction with the large cluster of Lys residues located at the protein surface opposite to the heme pocket (Fig. 1B). Future studies will address these issues.

## Materials and Methods

### Expression of NP7

The wt protein and its mutants were expressed, refolded, and purified as described previously^23^. SDS-PAGE assays indicated that protein preparations were estimated to be >95% pure. The expected molecular masses were confirmed by using MALDI TOF mass spectrometry (Voyager DE Pro, Applied Biosystems), confirming the presence of an initial Met-0 residue and two Cys–Cys disulphide bonds. Proteins were kept at −20 °C in 200 mM NaO Ac/HO Ac (Carl Roth), 10% (v/v) glycerol (Carl Roth) (pH 5.0) until use.

### X-ray crystallography

Protein crystals were obtained from 10 mg/ml NP7[E27V] solutions in 10 mM NaO*Ac*/HO*Ac* (pH 5.5) using the vapor-diffusion method upon mixing with an equal volume of crystallization solution (Hampton research) as indicated in Table S1^26,27^. Crystals were then soaked in the crystallization solution, containing 15% glycerol as a cryo-protectant. In the case of NP7[E27V](Imidazole) a 1 mM imidazole was added. Crystals were immediately frozen in liquid nitrogen and kept there until measurement. Diffraction data sets were collected at 100 K using the beamline BL14.2 at BESSYII (Helmholtz-Zentrum Berlin, Germany). The data set was processed with XDS^28^ and CCP4^29^. The molecular-replacement method was applied using MOLREP^26^ and an initial model from NP7 (PDB code 4XMC)^30^. Model building and refinement were carried out using WINCOOT^31^ and PHENIX^32^, respectively (see Table S1 for data collection and refinement statistics). PHENIX was used to check the stereochemical properties.

### Time-resolved spectroscopy

The experimental setups used for the femtosecond pump-probe and nanosecond photolysis studies were described previously^33–35^.

#### Ultrafast spectroscopy

A home-made optical parametric amplifier, driven by a regeneratively amplified Ti:sapphire laser (1 kHz repetition rate), provided excitation of the sample by a pump pulse centered at 530 nm, with 10 nm bandwidth. A broadband single-filament white-light continuum (WLC) generated in CaF_2_, was used as a probe pulse and covered a spectral range from 350 nm to 700 nm. After passing through a delay line, the pump pulse is then overlapped with the probe pulse on the sample. The transmitted probe light is dispersed on an optical multichannel analyzer (OMA) equipped with fast electronics, allowing single-shot recording of the probe spectrum at the full 1 kHz repetition rate. 2D maps of the differential transmission (ΔT/T) were collected as a function of probe wavelength and delay. The temporal resolution was estimated to be approximately 150 fs with a sensitivity better than 10^−4^ on the whole spectral range.

#### Nanosecond laser flash photolysis

The second harmonic (532 nm) of a nanosecond Q-switched Nd:YAG laser (Surelite II-10, Continuum) was used as the photolysis source of the CO complexes. The probe beam was a 75 W Xe arc lamp, and the transmitted light intensity was measured using a 5 stages photomultiplier (Applied Photophysics). The voltage was digitized with a digital oscilloscope (LeCroy LT374, 500 MHz, 4 GS s^−1^). The monitoring wavelength (436 nm) was selected by means of a monochromator (MS257 LOT-Oriel). Experiments were performed at 20 °C.

### Data analysis

Spectra acquired in the fs-ps time scale were analyzed by SVD, performed using the Matlab software as previously described^36^.

The procedure for merging the kinetics measured in the two time regimes was presented in a previous work^9^. Lifetime distributions associated with rebinding kinetics were determined using the program Memexp, based on a Maximum Entropy method^37,38^.

CO rebinding kinetics was analyzed with a reaction scheme reported in Scheme 1 and microscopic rate constants were retrieved by optimizing the numerical solutions of the associated differential equations to describe simultaneously the experimental data collected at two different CO concentrations. Numerical solutions were determined with the Matlab function ODE15s and optimized using the optimization package Minuit (CERN)^20,39^.

### Molecular modelling

The systems used in molecular simulations were modelled from the X-ray structures of NP7 solved at pH 5.8 (PDB ID 4XMC; resolution of 1.4 Å) and 7.8 (PDB ID 4XME; resolution of 1.3 Å). Two orientations were chosen for the E27Q mutant, which were generated by placing the amide nitrogen atom of Gln onto each of the carboxylate oxygen atoms of Glu27 (denoted as E27Q1 and E27Q2). Standard ionization states were assigned to all the protein residues. The only exception was Asp32, which was protonated at low pH and ionized at high pH^9,40,41^. Disulfide bridges between Cys5 and Cys124, and Cys42 and Cys173 were also introduced in the molecular systems^42^.

MD simulations were performed following the procedure reported previously for the wt NP7^9,25^. The charmm36^43,44^ force field was used for the protein residues and for the six-coordinated heme-bound CO (Fe(II)-CO) complex. The protein was placed in a pre-equilibrated cubic box (~70 Å per side) of TIP3P^42^ water molecules. Five and six chloride anions were added to keep the neutrality of the system for wt NP7 and its mutated variants, respectively. The systems were energy minimized using a multistep protocol that involved the separate optimization of hydrogen atoms, water molecules, and finally the whole system. Then, the systems were equilibrated by increasing the temperature in four steps (500 ps each) from 100 to 150, 200, 250 and to 300 K at constant volume. Then, an additional MD run (500 ps) at constant pressure (1 atm) and temperature (300 K) was run. At this point, production simulations (150 ns) were run in the NPT (1 atm, 300 K) ensemble using periodic boundary conditions and Ewald sums (grid spacing of 1 Å) for long-range electrostatic interactions. SHAKE and SETTLE algorithms were used to constrain bonds involving hydrogen atoms at their equilibrium bond lengths, and a time step of 2 fs for the integration of Newton’s equations. The analysis of the trajectories was performed for the ensemble of snapshots collected at 1 ps interval. All simulations were run using the NAMD program^45^.

Poisson-Boltzmann calculations were performed to estimate the shifts in pK_a_ between the wt NP7 and the mutated variants using the APBS program^46^. This approach has been amply used to analyze the pK_a_ values of titratable residues in proteins, affording a relevant saving in computational effort compared to other techniques^47^. Furthermore, we took advantage of the previous results reported by Di Russo *et al*.^13^ using constant pH MD simulations for NP4 for tuning present APBS calculations, particularly regarding the dielectric constant chosen for the interior of the protein (*ε*_*int*_). To this end, calculations were performed for a set of 50 snapshots taken from an independent MD simulation run for NP4 at low pH using different values of *ε*_*int*_ and grid density. Whereas the latter variable proved to have little influence on the results, the former had a drastic influence on the pK_a_ value of Asp30 (Asp32 in NP7), and the best agreement with the constant-pH MD results was found for *ε*_*int*_ = 20. This computational protocol was then used to determine the pK_a_ shifts for the NP7 mutated variants. Accordingly, calculations were performed for a set of 50 snapshots taken from the last 20 ns of the MD simulations. The snapshots were superposed through alignment of the backbone atoms of the protein, excluding the most flexible regions (AB, EF and GH loops, and three residues at N- and C-terminal regions). Atomic partial charges and radii were taken from the default parameters in the charmm36 force field, and a permittivity of 20 was assigned to the interior of the channel, whereas a value of 78.5 was used for the bulk environment. A focusing strategy was used for APBS calculations, with a finer grid of 0.25 Å/point.

## Electronic supplementary material

Supplementary Information

## References

[1] Champagne DE, Nussenzveig RH, Ribeiro JMC (1995). Purification, Partial Characterization, and Cloning of Nitric Oxide-carrying Heme Proteins (Nitrophorins) from Salivary Glands of the Blood-sucking Insect Rhodnius prolixus. J Biol Chem.

[2] Walker FA (2005). Nitric oxide interaction with insect nitrophorins and thoughts on the electron configuration of the {FeNO}6 complex. J Inorg Biochem.

[3] Kondrashov DA, Roberts SA, Weichsel A, Montfort WR (2004). Protein functional cycle viewed at atomic resolution: Conformational change and mobility in nitrophorin 4 as a function of pH and NO binding. Biochemistry.

[4] Andersen JF (2000). Kinetics and Equilibria in Ligand Binding by Nitrophorins 1: Evidence for Stabilization of a Nitric Oxide Ferriheme Complex through a Ligand-Induced Conformational Trap. Biochemistry.

[5] Ribeiro JM, Walker FA (1994). High affinity histamine-binding and antihistaminic activity of the salivary nitric oxide-carrying heme protein (nitrophorin) of Rhodnius prolixus. The Journal of Experimental Medicine.

[6] Falus, A, Grosman, N & Darvas, Z. *Histamine: biology and medical aspects*. SpringMed Pub. 359; Karger (2004).

[7] Heller A (2010). Electrochemistry and nictric oxide mass transport in cancer: Why ingestion of sodium nitrite could be effective in treating vascularized tumors. Phys Chem - Chem Phys.

[8] Flower DR, North ACT, Sansom CE (2000). The lipocalin protein family: structural and sequence overview. Biochim Biophys Acta, Protein Struct Mol Enzymol.

[9] Knipp M (2015). Structure and dynamics of the membrane attaching nitric oxide transporter nitrophorin 7 [v1; ref status: indexed]. F100 Research.

[10] Menyhard DK, Keserü GM (2005). Protonation state of Asp30 exerts crucial influence over surface loop rearrangements responsible for NO release in nitrophorin 4. FEBS Letters.

[11] Swails JM (2009). pH-Dependent Mechanism of Nitric Oxide Release in Nitrophorins 2 and 4. J Phys Chem B.

[12] Martí MA, Estrin DA, Roitberg AE (2009). Molecular basis for the pH dependent structural transition of nitrophorin 4. J Phys Chem B.

[13] Di Russo NV, Estrin DA, Marti MA, Roitberg AE (2012). pH-Dependent Conformational Changes in Proteins and Their Effect on Experimental pK_a_s: The Case of Nitrophorin 4. PLoS Comput Biol.

[14] Knipp M (2007). Spectroscopic and Functional Characterization of Nitrophorin 7 from the Blood-Feeding Insect Rhodnius prolixus Reveals an Important Role of Its Isoform-Specific N-Terminus for Proper Protein Function. Biochemistry.

[15] Andersen JF, Gudderra NP, Francischetti IMB, Valenzuela JG, Ribeiro JMC (2004). Recognition of anionic phospholipid membranes by an antihemostatic protein from a blood-feeding insect. Biochemistry.

[16] Knipp M, Zhang H, Berry RE, Walker FA (2007). Overexpression in *Escherichia coli* and functional reconstitution of the liposome binding ferriheme protein nitrophorin 7 from the blood sucking bug *Rhodnius prolixus*. Prot Expr Purif.

[17] Yang F, Zhang H, Knipp M (2009). A one-residue switch reverses the orientation of a heme b cofactor. Investigations on the ferriheme NO transporters nitrophorin 2 and 7 from the blood-feeding insect *Rhodnius prolixus*. Biochemistry.

[18] He C, Neya S, Knipp M (2011). Breaking the proximal FeII-NHis bond in heme proteins through local structural tension: Lessons from the heme b proteins nitrophorin 4, nitrophorin 7, and related site-directed mutant proteins. Biochemistry.

[19] Benabbas A (2010). Ultrafast Dynamics of Diatomic Ligand Binding to Nitrophorin 4. J Am Chem Soc.

[20] Abbruzzetti S (2012). Heterogeneous Kinetics of the Carbon Monoxide Association and Dissociation Reaction to Nitrophorin 4 and 7 Coincide with Structural Heterogeneity of the Gate-Loop. J Am Chem Soc.

[21] Goldbeck RA (2009). Optical Detection of Disordered Water within a Protein Cavity. J Am Chem Soc.

[22] Andersen JF, Montfort WR (2000). The Crystal Structure of Nitrophorin 2: a trifunctional antihemostatic protein from the saliva of *Rhodnius prolixus*. J Biol Chem.

[23] Shokhireva TK (2007). Assignment of the Ferriheme Resonances of the Low-Spin Complexes of Nitrophorins 1 and 4 by 1H and 13C NMR Spectroscopy: Comparison to Structural Data Obtained from X-ray Crystallography. Inorg Chem.

[24] Yang F (2009). A 1H and 13C NMR spectroscopic study of the ferriheme resonances of three low-spin complexes of wild-type nitrophorin 2 andnitrophorin2(V24E) as a function of pH. J Biol Inorg Chem.

[25] Oliveira A (2013). Kinetics and computational studies of ligand migration in nitrophorin 7 and its Δ1–3 mutant. Biochim Biophys Acta - Proteins and Proteomics.

[26] Ogata H, Knipp M (2012). Crystallization and preliminary X-ray analysis of the membrane-binding heme-protein nitrophorin 7 from *Rhodnius prolixus*. Acta Crystallographica F.

[27] Benvenuti M, Mangani S (2007). Crystallization of soluble proteins in vapor diffusion for x-ray crystallography. Nat Protocols.

[28] Kabsch W (1993). Automatic processing of rotation diffraction data from crystals of initially unknown symmetry and cell constants. J Appl Cryst.

[29] Project CC (1994). The CCP4 suite: Programs for protein crystallography. Acta Crystallographica Sect D.

[30] He C, Ogata H, Knipp M (2010). Formation of the complex of nitrite with the ferriheme b β-barrel proteins nitrophorin 4 and nitrophorin 7. Biochemistry.

[31] Emsley P, Cowtan K (2004). Coot: model-building tools for molecular graphics. Acta Crystallographica Sect D.

[32] Adams PD (2011). The Phenix software for automated determination of macromolecular structures. Methods.

[33] Polli, D, Laer, L & Cerullo, G. High-time-resolution pump-probe system with broadband detection for the study of time-domain vibrational dynamics. *Rev Sci Instrum* **78**(10), 103–108 (2007). 10.1063/1.2800778 17979407

[34] Abbruzzetti S (2006). Time-resolved methods in Biophysics. 2. Monitoring haem proteins at work with nanosecond laser flash photolysis. Photochem Photobiol Sci.

[35] Bruno S (2007). Different roles of protein dynamics and ligand migration in non-symbiotic hemoglobins AHb1 and AHb2 from *Arabidopsis thaliana*. Gene.

[36] Henry, E. R. & Hofrichter, J. Singular value decomposition: application to analysis of experimental data. In: *Numerical computer methods* (eds Brand, L. & Johnson, M. L.). Academic Press, Inc (1992).

[37] Steinbach PJ (2002). Inferring Lifetime Distributions from Kinetics by Maximizing Entropy Using a Bootstrapped Model. J Chem Inf Comput Sci.

[38] Steinbach PJ, Ionescu R, Matthews CR (2002). Analysis of Kinetics Using a Hybrid Maximum-Entropy/Nonlinear-Least-Squares Method: Application to Protein Folding. Biophys J.

[39] Abbruzzetti S, Spyrakis F, Bidon-Chanal A, Luque FJ, Viappiani C (2013). Ligand migration through hemeprotein cavities: insights from laser flash photolysis and molecular dynamics simulations. Phys Chem Chem Phys.

[40] Marti MA, Lebrero MCG, Roitberg AE, Estrin DA (2008). Bond or Cage Effect: How Nitrophorins Transport and Release Nitric Oxide. J Am Chem Soc.

[41] Maes EM, Weichsel A, Andersen JF, Shepley D, Montfort WR (2004). Role of Binding Site Loops in Controlling Nitric Oxide Release: Structure and Kinetics of Mutant Forms of Nitrophorin 4. Biochemistry.

[42] Knipp M, Taing JJ, He C (2011). Reduction of the lipocalin type heme containing protein nitrophorin. Sensitivity of the fold-stabilizing cysteine disulfides toward routine heme-iron reduction. J Inorg Biochem.

[43] Huang J, MacKerell AD (2013). CHARMM36 all-atom additive protein force field: Validation based on comparison to NMR data. J Comput Chem.

[44] Best RB (2012). Optimization of the Additive CHARMM All-Atom Protein Force Field Targeting Improved Sampling of the Backbone ϕ, ψ and Side-Chain χ_1_ and χ_2_ Dihedral Angles. J Chem Theory Comput.

[45] Phillips JC (2005). Scalable molecular dynamics with NAMD. J Comput Chem.

[46] Baker NA, Sept D, Joseph S, Holst MJ, McCammon JA (2001). Electrostatics of nanosystems: Application to microtubules and the ribosome. Proc Natl Acad Sci USA.

[47] Ren P (2012). (2012). Quarterly Reviews of Biophysics,. Biomolecular electrostatics and solvation: A computational perspective. Quart Rev Biophys.

