# Supplementary material for: Electrostatic Tuning of the Ligand Binding Mechanism by Glu27 in Nitrophorin 7
Source: Sci Rep. 2018 Jul 18;8:10855. doi: 10.1038/s41598-018-29182-3 (PMC6052033; doi:10.1038/s41598-018-29182-3)
Supplement: Supplementary file 1 — Supplementary Information [file 41598_2018_29182_MOESM1_ESM.docx]

**Supporting Information**

**Electrostatic Tuning of the Ligand Binding Mechanism by Glu27 in Nitrophorin 7**

Stefania Abbruzzetti,^1,*^ Alessandro Allegri,^1^ Axel Bidon-Chanal,^2^ Hideaki Ogata,^3,§^ Giancarlo Soavi,^4^ Giulio Cerullo,^5^ Stefano Bruno,^6^ Chiara Montali,^1^ F. Javier Luque,^2,*^ and Cristiano Viappiani^1,*^

^1^ Dipartimento di Scienze Matematiche, Fisiche e Informatiche, Università degli Studi di Parma, Parco Area delle Scienze 7/A, 43124 Parma, Italy

^2^ Department of Nutrition, Food Sciences and Gastronomy, Faculty of Pharmacy and Food Sciences and Institute of Biomedicine (IBUB), University of Barcelona, Avda. Prat de la Riba 171, Santa Coloma de Gramenet, Spain

^3^ Max-Planck Institute for Chemical Energy Conversion, Stiftstrasse 34-36, D-45470 Mülheim an der Ruhr, Germany

^4^ Cambridge Graphene Centre, University of Cambridge, 9 JJ Thomson Avenue, Cambridge CB3 OFA, U.K

^5^ IFN-CNR, Dipartimento di Fisica, Politecnico di Milano, Piazza Leonardo da Vinci 32, 20133 Milano, Italy

^6^ Dipartimento di Scienze degli Alimenti e del Farmaco, Università degli Studi di Parma, Parco Area delle Scienze 27/A, 43124 Parma, Italy

^§^ Present address: Institute of Low Temperature Science, Hokkaido University
Kita19-Nishi8, Kita-ku, 060-0819 Sapporo, Japan. Email: hideaki.ogata@pop.lowtem.hokudai.ac.jp

^*^ Corresponding author: S.A. (email: stefania.abbruzzetti@unipr.it), F.J.L. (email: fjluque@ub.edu) or C.V. (email: cristiano.viappiani@unipr.it)

**Index**

Table S1 S3

Table S2 S4

Table S3 S5

FigureS1 S7

FigureS2 S8

Figure S3 S9

Figure S4 S10

Figure S5 S11

Figure S6 S12

**Table S1** Data collection and refinement statistics for the aquo form of ferric NP7(E27V) protein, and to the complex with heme-bound imidazole (IMH).

|  | NP7(E27V)^a^ | NP7(E27V)IMH ^b^ |
| --- | --- | --- |
| ***Data collection*** |  |  |
| X-ray source | BESSYII  BL14.2 | BESSYII  BL14.2 |
| Wavelength (Å) | 0.91841 | 0.91841 |
| Space group | *P*2_1_ | *P*2_1_ |
| Unit-cell parameters  a (Å)  b (Å)  c (Å)  β(°) | 38.23  66.89  38.71  116.6 | 38.26  66.64  38.49  116.7 |
| Resolution (Å) | 34.63-1.70 (1.74-1.70) | 30.56-1.60 (1.64-1.60) |
| No. of observed reflections | 72215 | 83100 |
| No. of unique reflections | 18666 | 22579 |
| *R*_merge_ | 0.057 (0.488) | 0.058 (0.581) |
| Completeness (%) | 96.6 (95.3) | 98.7 (91.0) |
| <*I/σ*(*I*)> | 17.6 (3.0) | 13.4 (1.9) |
| ***Refinement*** |  |  |
| Resolution (Å) | 34.6-1.7 | 30.6-1.6 |
| *R* (%) | 16.1 | 15.9 |
| *R*_free_ (%) | 21.3 | 21.2 |
| No. of residues | 184 | 184 |
| No. of water molecules | 148 | 121 |
| Rmsd bond length (Å) | 0.006 | 0.006 |
| Rmsd bond angle (°) | 1.057 | 1.075 |
| Ramachandran plot  Outliers (%)  Favored (%) | 0.0  98.91 | 0.0  98.91 |
| Average B  Protein (Å^2^)  Ligand heme (Å^2^)  Ligand (Å^2^)  Solvent (Å^2^) | 22.4  22.2  25.4  27.4 | 24.0  23.7  21.4  31.2 |
| Heme orientation | B | B |
| PDB entry | 5M6J | 5M6K |

a) 0.33% w/v anthrone, 0.33% w/v Congo Red, 0.33% w/v N-(2-acetamido)-2-aminoethanesulfonic acid, 0.02M HEPES sodium pH 6.8, b) 0.25% w/v hexamminecobalt(III) chloride, 0.25% w/v salicylamide, 0.25% w/v Sulfanilamide, 0.25% vanillic acid, 0.02M HEPES sodium pH 6.8.

**Table S2** RMSD (Å) of the energy-minimized average structures taken from the trajectories run for wt NP7 (in closed, WTc, and open, WTo, forms) and its mutated (E27Q, E27V) variants. The average structure was derived from the snapshots sampled in the last 20 ns of the MD simulations. The RMSD values were computed for the backbone atoms, excluding the first three residues at the N-terminus and last four residues at the C-terminus (plain text), and upon exclusion of residues in loops AB (31-41), EF (91-104) and GH (127-135) (italics).

|  | E27Q1 | E27Q2 | E27V  (**A**-heme) | E27V  (**B**-heme) | WTo |
| --- | --- | --- | --- | --- | --- |
| WTc | 1.7  *1.1* | 1.4  *1.2* | 2.0  *1.3* | 1.4  *1.0* | 2.4  *1.8* |
| E27Q1 |  | 1.7  *1.1* | 1.9  *1.2* | 1.6  *1.0* | 2.8  *2.0* |
| E27Q2 |  |  | 1.9  *1.1* | 1.4  *1.0* | 2.3  *1.7* |
| E27V  (**A**-heme) |  |  |  | 1.9  *1.0* | 3.1  *2.0* |
| E27V  (**B**-heme) |  |  |  |  | 2.4  *1.7* |

**Table S3** Dependence of the population (%) of the neutral and deprotonated species of NP4, NP7, NP7(E27V) and NP7(E27Q) on conformational equilibrium between closed and open species at pH 5.5 and pH 7.5.

| K_D_ / K_H_ | pH | [NPX-O^0^] | [NPX-O^-^] | [NPX-C^0^] | [NPX-C^-^] |
| --- | --- | --- | --- | --- | --- |
| **NP4** | | | | | |
| 1000 / 1.6 × 10^-2^  (6.3 × 10^-3^) | 5.5 | 1.1  (3.1) | 28.1  (48.4) | 70.7  (48.4) | 0.0  (0.0) |
|  | 7.5 | 0.0  (0.0) | 97.4  (98.9) | 2.4  (1.0) | 0.1  (0.1) |
| 100 / 1.6 × 10^-3^  (6.3 × 10^-3^) | 5.5 | 0.2  (0.6) | 3.8  (9.0) | 96.0  (90.3) | 0.0  (0.1) |
|  | 7.5 | 0.0  (0.1) | 79.3  (90.0) | 19.9  (9.0) | 0.8  (0.9) |
| 10 / 1.6 × 10^-4^  (6.3x 10^-4^) | 5.5 | 0.0  (0.1) | 0.4  (1.0) | 99.5  (98.9) | 0.0  (0.1) |
|  | 7.5 | 0.0  (0.0) | 27.7  (47.6) | 69.5  (47.6) | 2.8  (4.8) |
| 1 / 1.6 ×10^-5^  (6.3 × 10^-5^) | 5.5 | 0.0  (0.0) | 0.0  (0.1) | 99.9  (99.8) | 0.0  (0.1) |
|  | 7.5 | 0.0  (0.0) | 3.7  (8.3) | 92.6  (83.3) | 3.7  (8.3) |
| **wt NP7** | | | | | |
| 1000 / 1.6 | 5.5 | 3.7 | 93.8 | 2.4 | 0.1 |
|  | 7.5 | 0.0 | 99.8 | 0.0 | 0.1 |
| 100 / 1.6 × 10^-1^ | 5.5 | 3.1 | 76.9 | 19.3 | 0.8 |
|  | 7.5 | 0.0 | 98.7 | 0.2 | 1.0 |
| 10 / 1.6 × 10^-2^ | 5.5 | 1.1 | 27.4 | 68.8 | 2.7 |
|  | 7.5 | 0.0 | 88.8 | 2.2 | 8.9 |
| 1 / 1.6 × 10^-3^ | 5.5 | 0.1 | 3.7 | 92.5 | 3.7 |
|  | 7.5 | 0.0 | 44.4 | 11.2 | 44.4 |
| **NP7(E27V)** | | | | | |
| 1000 / 5.0 | 5.5 | 3.8 | 95.4 | 0.8 | 0.1 |
|  | 7.5 | 0.0 | 99.9 | 0.0 | 0.1 |
| 100 / 5.0 × 10^-1^ | 5.5 | 3.5 | 88.6 | 7.0 | 0.9 |
|  | 7.5 | 0.0 | 98.9 | 0.1 | 1.0 |
| 10 / 5.0 × 10^-2^ | 5.5 | 2.1 | 51.7 | 41.1 | 5.2 |
|  | 7.5 | 0.0 | 90.2 | 0.7 | 9.0 |
| 1 / 5.0 × 10^-3^ | 5.5 | 0.4 | 10.0 | 79.6 | 10.0 |
|  | 7.5 | 0.0 | 48.1 | 3.8 | 48.1 |
| **NP7(E27Q)** | | | | | |
| 1000 / 25.1 | 5.5 | 3.8 | 95.9 | 0.2 | 0.1 |
|  | 7.5 | 0.0 | 99.9 | 0.0 | 0.1 |
| 100 / 2.5 × 10^-1^ | 5.5 | 3.7 | 93.8 | 1.5 | 0.9 |
|  | 7.5 | 0.0 | 99.0 | 0.0 | 1.0 |
| 10 / 2.5 × 10^-2^ | 5.5 | 3.1 | 77.0 | 12.2 | 7.7 |
|  | 7.5 | 0.0 | 90.7 | 0.1 | 9.1 |
| 1 / 2.5 × 10^-3^ | 5.5 | 1.1 | 27.6 | 43.7 | 27.6 |
|  | 7.5 | 0.0 | 49.6 | 0.8 | 49.6 |

The population of protonated and deprotonated forms of closed and open species of NP4 and NP7 was determined using Eqs. S1-S4, following the formalism presented in ref. 10 in the manuscript.

Note that the term 'K_D_[H^+^]' in Eq. S1 was incorrectly written as 'K_D_' in the expression given for [NPX-O^0^] in the Supplementary Material of ref. 10.

$\text{[NPX-O}^{0}]\text{=}\frac{\text{K}_{\text{D}}[\text{H}^{+}]}{\text{K}_{\text{a}}^{\text{O}}\left[1+\text{K}_{\text{D}}+[\text{H}^{+}]\left(\frac{\text{K}_{\text{D}}}{\text{K}_{\text{a}}^{\text{O}}}+\frac{1}{\text{K}_{\text{a}}^{\text{C}}}\right)\right]}$ (S1)

$\text{[NPX-O}^{-}]\text{=}\frac{\text{K}_{\text{D}}}{1+\text{K}_{\text{D}}+[\text{H}^{+}]\left(\frac{\text{K}_{\text{D}}}{\text{K}_{\text{a}}^{\text{O}}}+\frac{1}{\text{K}_{\text{a}}^{\text{C}}}\right)}$ (S2)

$\text{[NPX-C}^{0}]\text{=}\frac{[\text{H}^{+}]}{\text{K}_{\text{a}}\left[1+\text{K}_{\text{D}}+[\text{H}^{+}]\left(\frac{\text{K}_{\text{D}}}{\text{K}_{\text{a}}^{\text{O}}}+\frac{1}{\text{K}_{\text{a}}^{\text{C}}}\right)\right]}$ (S3)

$\text{[NPX-C}^{-}]\text{=}\frac{1}{1+\text{K}_{\text{D}}+[\text{H}^{+}]\left(\frac{\text{K}_{\text{D}}}{\text{K}_{\text{a}}^{\text{O}}}+\frac{1}{\text{K}_{\text{a}}^{\text{C}}}\right)}$ (S4)

**Figure S1.** Results of global analysis of the complete course of CO binding kinetics to wt NP7 (A: pH=7.5, B: pH=5.5; data adapted from ref. 7), NP7(E27V) (C: pH=7.5, D: pH=5.5), NP7(E27Q) (E: pH=7.5, F: pH=5.5), at T=20°C and 1 (black) and 0.1 atm (gray). The fits (yellow lines) are superimposed to the experimental data. The time course of other relevant species shown in Scheme 1 is reported: DP (black), T_2_ (blue), T_3_ (cyan), T_4_ (magenta), NP (red) and NP* (green).





**Figure S2.** Chemical numbering and schematic representation of the **A** and **B** orientations of the heme. Spheres denote the position of apolar residues in the heme pocket of *R. prolixus* NP7 that enclose the ligand at the distal side (adapted from Figure S2 in ref. 17).


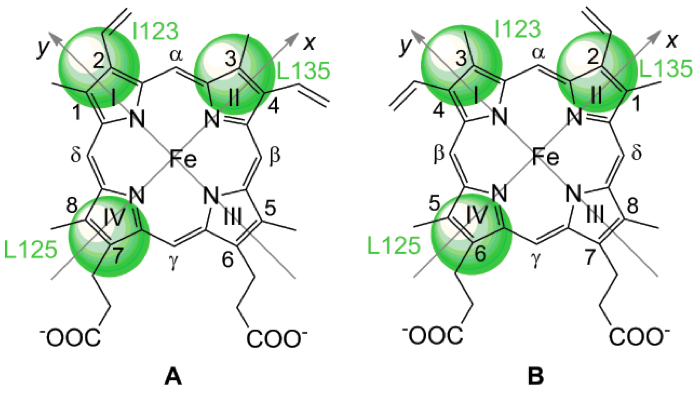


The 'orientational heterogeneity' of the heme cofactor is known to be a structural feature of b-type hemoproteins.^1^ Solution 1H NMR studies for sperm whale myoglobin revealed that there are two slowly interconverting species at equilibrium, which differ by a 180^o^-rotation about the α-γ-meso axis.^2^ The protein folding is essentially the same for the two orientations, as noted in similar proton NMR spectra. The equilibration between the two orientations requires the rupture of the proximal histidine-heme bond, leading to partial dissociation that frees the cofactor from the heme pocket, and reorientation of the cofactor without leaving the protein cage.^2,3^

1 Y. Kanaki, A. Harada, T. Shibata, R. Nishimura, K. Namili, M. Watanabe, S. Nakamura, F. Yumoto, T. Senda, A. Suzuki, S. Neya and Y. Yamamoto, *Biochemistry* 2017, **56**, 4500–4508.

2 G. N. La Mar, H. Toi and R. Krishnamoorthi, *J. Am. Chem. Soc*. 1984, **106**, 6395–6401.

3 J. B. Hauksson, G. N. La Mar, U. Pande, R. K. Pandey, D. W. Parish, J. P. Singh and K. M. Smith, *Biochim. Biophys. Acta* 1990, **1041**, 186–194.

**Figure S3.** Arrangement of the heme and selected residues (Glu27, Thr168, and Tyr30) after superposition of the protein backbone in 11 snapshots taken every 5 ns in the last part of the MD simulation for wt NP7, E27V (A-heme), and E27Q (Q1).

**
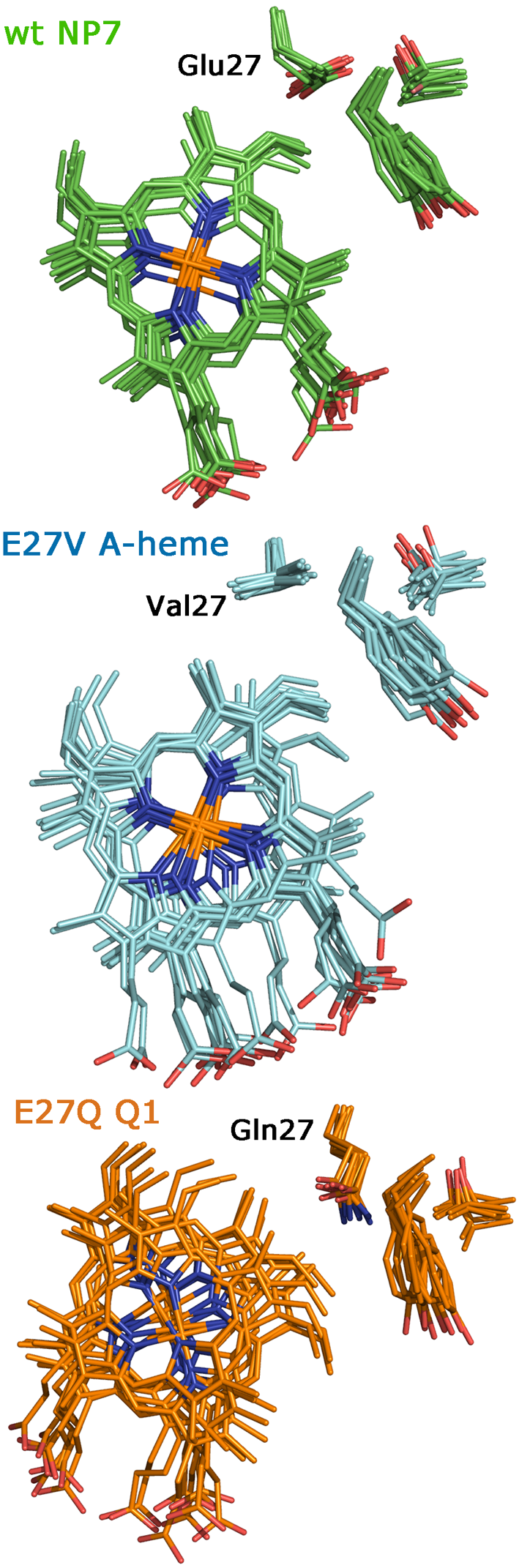
**

The rearrangements observed in the heme occurred at the beginning of the trajectory and remained stable along the rest of the simulation. This is shown for a set of 11 snapshots taken regularly every 5 ns in the last 50 ns of the trajectory. Only small fluctuations around the average structure were observed, this behaviour being found in all cases.

**Figure S4.** Superposition of the heme moiety and selected residues in the MD energy-minimized average structures. Comparison of the relative position of the heme and side chains of Ile123, Leu125, Ile132 and Leu135 in the average structures of wt NP7 (green-colored C atoms) and (A) NP7(E27V) with **A**-heme (light blue-colored C atoms), (B) NP7(E27V) with **B**-heme (dark blue-colored Catoms), (C) NP7(E27Q1) (orange-colored Catoms), and (C) NP7(E27Q2) (magenta-colored C atoms). The heme-bound CO is shown as spheres.


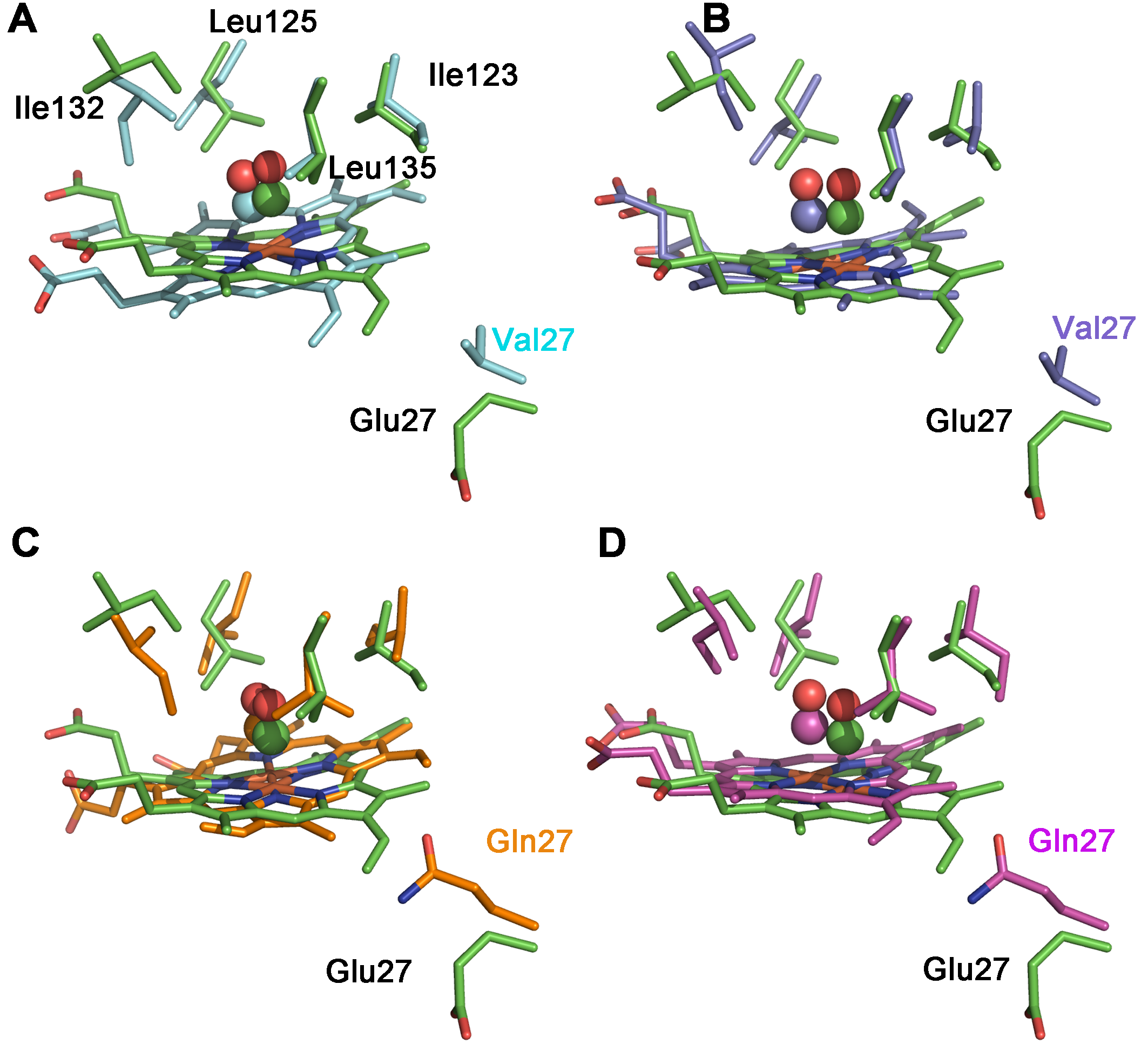


**Figure S5.** (A) Hydrogen-bond interactions of Glu27 with Thr168, Tyr175, and Phe43 found in the MD simulation of the closed state of wt NP7. Average distances of hydrogen-bond contacts are shown in bold. (B) Comparison of the spatial location of Glu27, Thr168, Tyr145, Phe43, and Lys143 in the MD simulation (carbon atoms in green) and in the X-ray structure (PDB ID 4XMC; carbon atoms in white). Note the distinct arrangement observed for this latter residue in the MD simulation and in the X-ray structure.

**
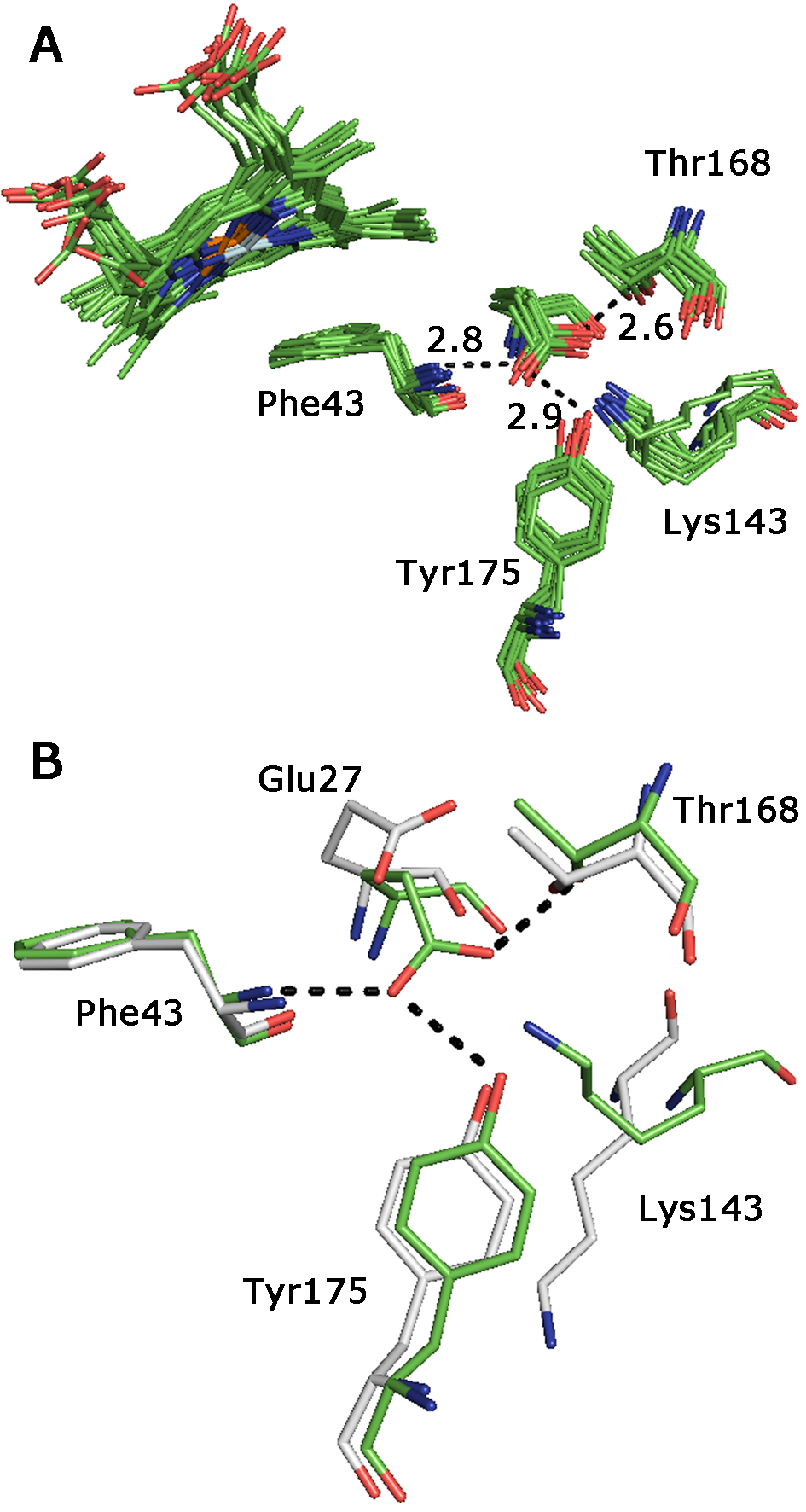
**

Along the MD simulation, the side chain slightly rearranges to form hydrogen-bond interactions with the backbone NH group of Phe43, and the hydroxyl groups of Thr168 and Tyr175, which roughly maintain the arrangement found in the X-ray structure. Moreover, the side chain of Lys143, located in the surface of the protein, reorients to provide electrostatic stabilization.

**Figure S6.** Comparison of the spatial location of Glu27, Thr168, Tyr145, Phe43, and Lys143 in the MD simulation of the open state of wt NP7 (carbon atoms in green; snapshots taken regularly along the last 50 ns of the simulation reported in ref. 25). For the sake of clarity, only the backbone skeleton of a single structure is shown.

**
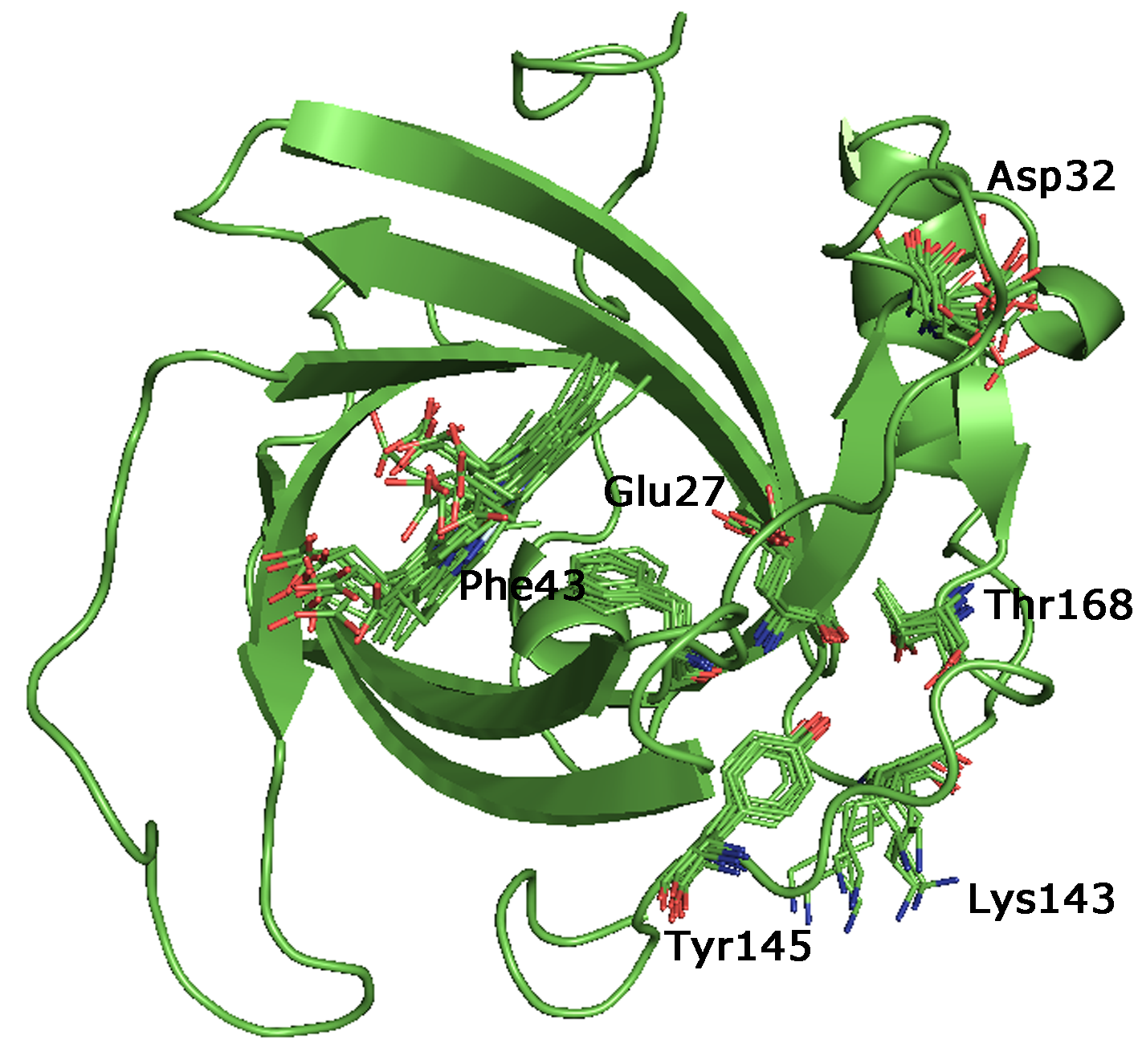
**

Breakage of the hydrogen-bond interaction between Asp32 and Ile132 promotes the opening of the loop AB, which adopts a variety of conformational states. Asp32 becomes well exposed to the aqueous solvent. Furthermore, Glu27 adopts an extended conformation, resulting from the relief of the steric stress in the closed species, and becomes more exposed to water molecules.
